# Integrated Evaluation Method of the Health-Related Physical Environment in Urbanizing Areas: A Case Study From a University Campus in China

**DOI:** 10.3389/fpubh.2022.801023

**Published:** 2022-02-08

**Authors:** Yizhou Wu, Siqin Wu, Xiaoli Qiu, Shuai Wang, Shenyi Yao, Wentao Li, Heyuan You, Jinrong Zhang, Shuyi Xia, Yufei Guo

**Affiliations:** ^1^School of Design and Architecture, Zhejiang University of Technology, Hangzhou, China; ^2^School of Public Administration, Zhejiang University of Finance and Economics, Hangzhou, China

**Keywords:** health-related physical environment, integrated evaluation, priority intervention area, university campus, China

## Abstract

Environmental deterioration in urbanizing areas increases the risks of sudden death as well as chronic, infectious, and psychological diseases. Quantifying health-related physical environment can assess the health risk of urban residents. This study uses an integrated evaluation method to simulate the health-related physical environment in the four dimensions of acoustic, wind, thermal, and landscape. According to the case study of one university campus in an urbanizing area in China, results show that (1) areas with unqualified equivalent A sound levels are generally the sports area, green square 1 and laboratory areas, and residents who stay in these areas for a long time suffer the risks of hearing loss and mental stress. (2) The windless area ratio of teaching area 1 and dormitory area 4 is larger than 20%, and respiratory health risks increase because these areas relate to relatively wind discomfort. (3) The high-temperature zone ratio of sports area and green square 2 is larger than 50%, and heatstroke risks increase since these areas relate with low thermal comfort. (4) The overall landscape perception level of dormitories and dining areas is lower than that of the teaching area, and it can cause anxiety and irritability. (5) The sports area has the lowest average overall score of the health-related physical environment among all functional areas, followed by laboratory areas. These findings indicate that the proposed model and method can be valuable tools for the pre-evaluation and optimization of urban planning. It can reduce the health risks of residents in urbanizing areas and can benefit residents' health and urban sustainable development.

## Introduction

Rapid urbanization directly affects and changes the physical environment of urbanizing areas, leading to a series of outstanding environmental problems, such as the heat island effect, noise pollution, and air pollution. Environmental deterioration increases the risks of sudden death, chronic diseases, infectious diseases, and psychological diseases. An arduous and important task faced by urban planners is to find ways to optimize the urban form, create urban space and provide people with a high-quality life. The urban physical environment includes wind, thermal, acoustic, landscape, and other elements. The state of the health-related physical environment directly affects the overall well-being of the city and its residents. Previous research revealed that a low-quality acoustic environment can result in tinnitus and hearing loss (1); ventilation environment affects the characteristics of urban air flow and also indirectly affects people's respiratory health; the deterioration of urban thermal environment not only reduces people's thermal comfort (2) but also increase the risks of sunburn and heatstroke (3, 4); landscape results in different emotions, such as happiness, relaxation, excitement, depression, anxiety, sadness, etc. Providing good comfort is considered an important path to deal with the environmental health problem. Therefore, various environmental factors that affect public health in urbanizing areas should be comprehensively analyzed.

Existing studies on the physical environment in urban planning and design generally include post-use evaluation and in-design evaluation (5). The post-occupancy evaluation examines the effectiveness of the residential environment in use (6), including evaluation of building energy consumption (7), residential environment satisfaction (8), and physical environmental impact (9, 10). This method measures the data collected to evaluate part of or the whole city after it is in use. In-design evaluation quantifies and predicts the performance of the design-based physical environment through calculation and simulation models. The obtained results and evaluations are often used to guide design optimization and adjustment. Previous research focused on the post-fact evaluation of the physical environment, but in-design evaluation also should be analyzed to predict the performance of the physical environment that has an important impact on public health. Therefore, pre-evaluation is designed to evaluate the health-related physical environment in urbanizing areas in this study.

Extensive research has been carried out to determine the influence of the thermal environment. Le Corbusier pioneered the use of shading and ventilation as the basic strategy of urban architectural design (11). This method incorporates climate into the scope of urban planning and design. Olgyay (12) argued that sustainable design should systematically integrate design, location, climate, and human comfort. In recent years, the use of numerical simulation to explore the thermal comfort of the urban thermal environment has attracted extensive attention (13, 14). Thermal comfort relates to temperature, wind speed, relative humidity, and thermal radiation (15–17). Indices for measuring the comfort degree of thermal environment include predicted mean vote (PMV) (18), physiological equivalent temperature (PET) (19), and universal thermal comfort index (UTCI) (20). Research of the acoustic environment focuses on the influence of noise on human hearing health (21, 22), methods of reducing noise influence (23), and application of noise reduction measures (24). The equivalent A sound level (LAeq) and the peak traffic noise (L10) are often used as the evaluation indices of the noise prediction model (25, 26). Previous studies revealed that green landscape has a positive effect on human health (27). Research on health-related physical environment focuses on the thermal environment and air quality. However, the health-related physical environment tightly relates with acoustic, wind, thermal, and landscape (28, 29). Meanwhile, few researchers have paid attention to the subjective perception of the health-related physical environment, especially visual quality (30).

This research mainly aims to use the in-design evaluation method to identify priority intervention areas where the health-related physical environment can be optimized and improved and provide a new approach for pre-evaluation and optimization in urban planning and design. Specifically, this study is divided into the following parts. (1) The technical protocol and research method are determined. The wind, thermal and acoustic environment are calculated and simulated using software, the level of landscape perception is determined through questionnaire surveys, the simulation results are analyzed based on evaluation indices, the environment of different dimensions is quantified and graded based on evaluation standards and spatial raster analysis is conducted with GIS technology. (2) The health-related physical environment of Zhejiang University of Technology Zhaohui campus is simulated using the combination of subjective and objective methods. The four types of the health-related physical environment of wind, thermal, acoustic, and landscape at the pedestrian level are studied and comprehensively evaluated through the combination of objective simulation results and subjective quantitative grading. (3) The key conclusions of this research, the applicability of the method and the possible future research interests are provided.

## Research Framework

### Study Area

The Zhaohui campus of Zhejiang University of Technology is located in Hangzhou City in China and covers an area of 597,000 m^2^. The summer is hot and humid with prevailing southeast wind, and the climate is typical humid subtropical. The whole area, which is surrounded by roads (elevated roads to the west and the north), contains nearly all factors that affect the health-related physical environment of campus, including elevated roads, trees, rivers, and high-rise buildings outside the school.

**Figure 1:**
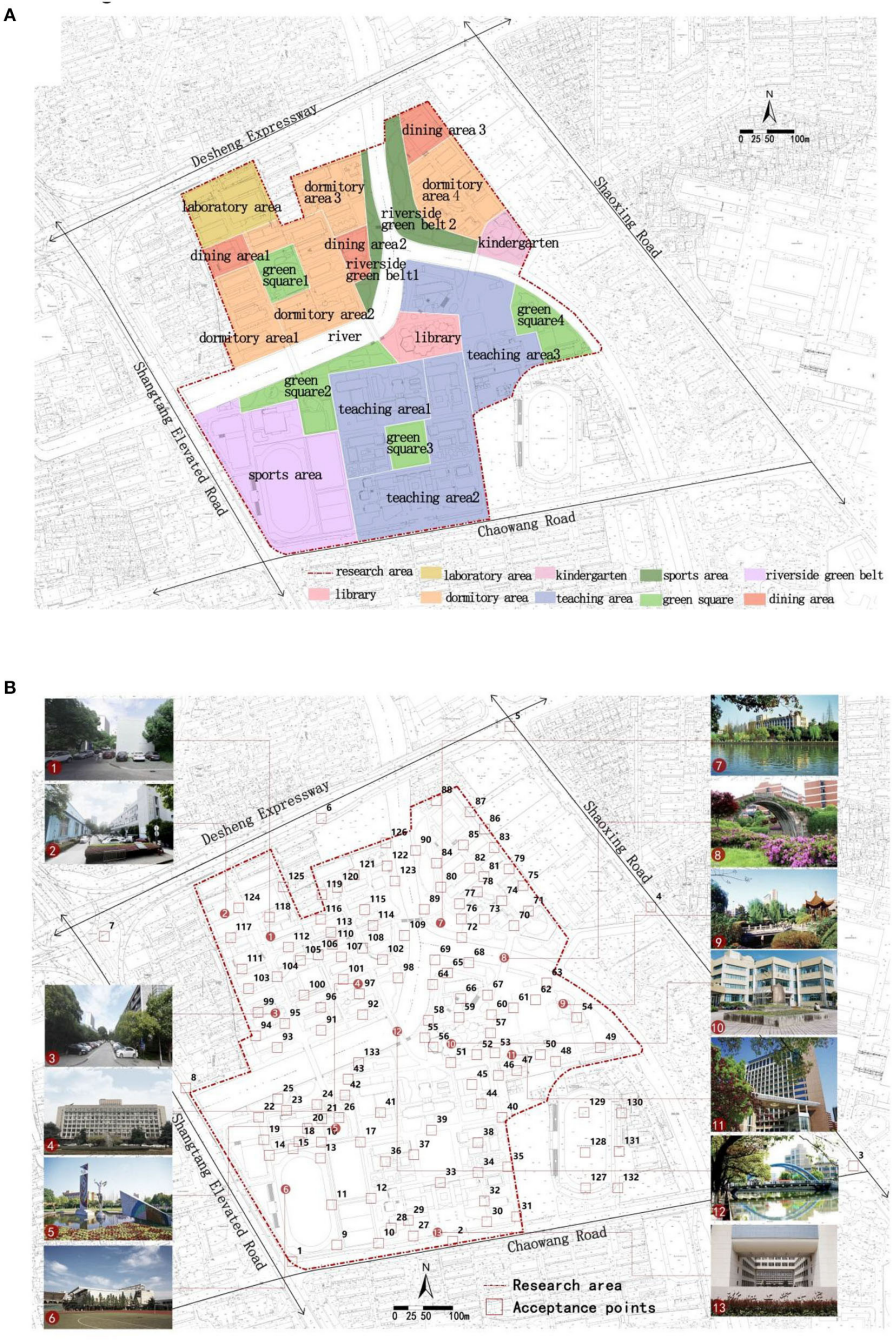
Zhaohui Campus of Zhejiang University of Technology: **(A)** map of functional zones, **(B)** acceptance point layout and current situation.

A functional area identifier is used to represent the function of each of the different functional areas (Figure 1A). Areas are divided according to the types of activities, and spatial forms in this area and are divided into 20 areas, including dormitories, sports area, laboratory areas, dining areas, riverside green belt, green squares and teaching areas. Although some activities are generated outside the boundary of an area, a subjective impact is still exerted on people in the area. Therefore, the environment outside the boundary is also included in the simulation, and a typical affected area will be described by a specific method. However, the principle of functional area division evaluation is to be within walking distance.

### Technology Route

**Figure 2:**
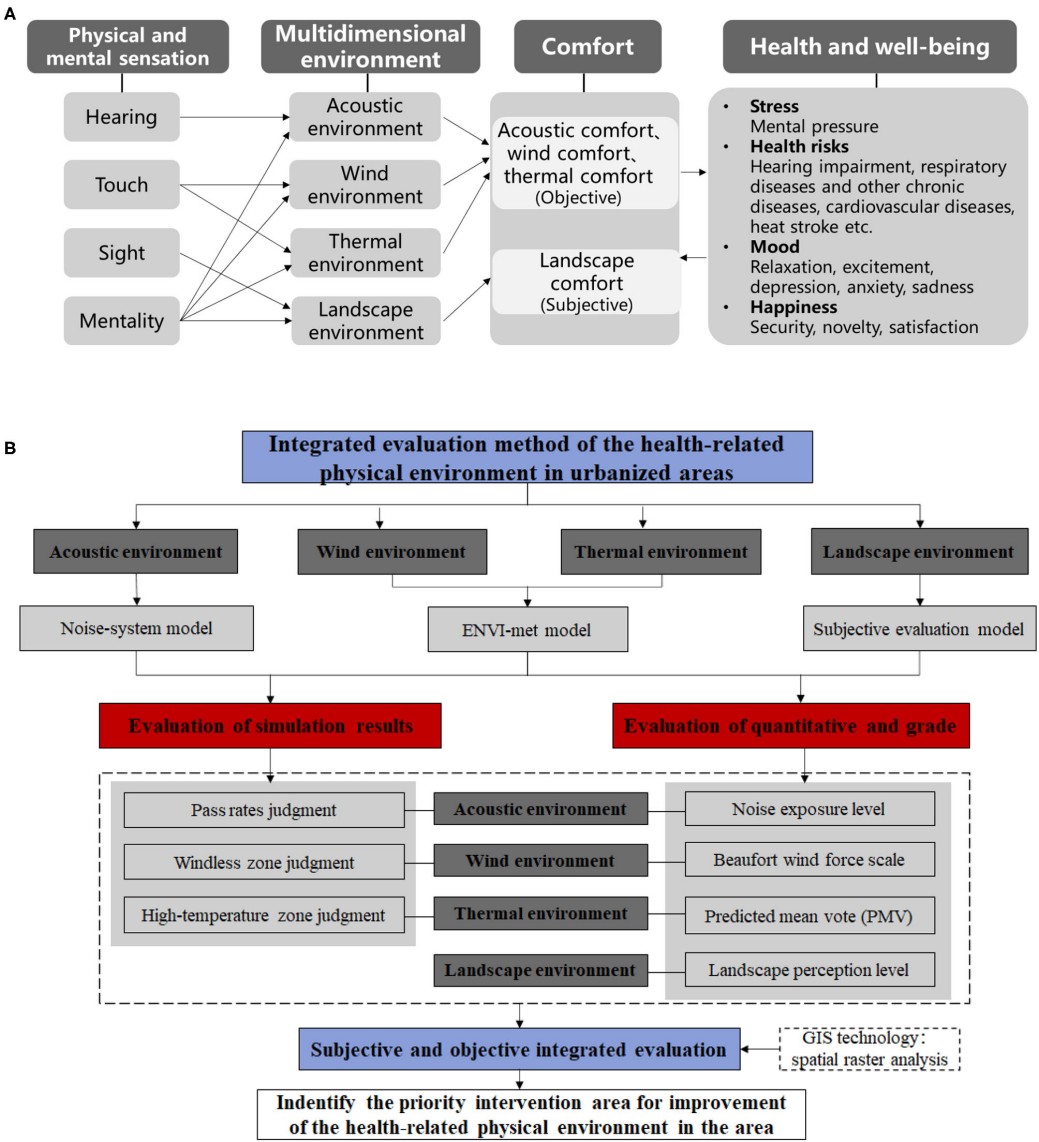
Research framework: **(A)** conceptual framework for health-related physical environment, **(B)** technology route.

The physical environment can be perceived through physical and mental sensations. The physical and mental sensation includes hearing, touch, sight, and mentality (31). The physical and mental sensation can obtain information about the acoustic, wind, thermal, and landscape environments. The multidimensional environment tightly relates to human health and well-being because it impacts acoustic comfort, wind comfort, thermal comfort, and comfort. Therefore, the conceptual framework for the health-related physical environment is shown in Figure 2A.

The technical route is shown in Figure 2B. This work studies the four types of the health-related physical environment of acoustic, wind, thermal, and landscape. First, the wind, thermal and acoustic environments are modeled using simulation methods, and the subjective evaluation method is built for the landscape environment by using questionnaire surveys. Second, the simulation results of acoustic, wind, and thermal environments are analyzed using different evaluation indices. In addition, the equivalent A sound level, wind speed, PMV value, and landscape preference value of the receiving stations are measured, and the health-related physical environment of each dimension is quantified into scores according to different evaluation standards. Finally, the spatial raster analysis is performed with ArcGIS software to determine priority intervention areas, where the health-related physical environment can be improved.

## Methods

### Numerical Simulation

This study uses the calculation model of outdoor sound propagation attenuation in the Chinese standard Technical Guidelines for Environmental Impact Assessment-Acoustic Environment (HJ2.4-2009) for acoustic environment simulation and NoiseSystem software developed by Huan'an Technology Co., Ltd. The specific model calculation method is introduced in the study of Wu et al. (32). This study will not go into details. The software considers the comprehensive effect of all sound sources, attenuators, and meteorological elements during sound propagation in the prediction area and uses the output equivalent A sound level (LA), octave band sound pressure level (LP), and other data to evaluate the sound environment.

The ENVI-met model developed by Professor Michael Bruse of Germany is used to establish the microscale ENVI-met model for wind and thermal environment simulation (33). It can be used to study the microscale numerical simulation of the interaction of the surface, vegetation and air in the urban area. It can also be used to analyze the impact of small-scale changes on the microclimate in urban design. ENVI-met usually calculates and outputs meteorological data, such as air temperature, humidity, wind speed and average radiation temperature. At the same time, the BioMet module can be used to calculate PMV. The setting of PMV parameters is based on general Hangzhou residents, with the walking speed set to 3.6 km/h (1 m/s) and the insulation coefficient of summer clothes set to 0.5 clo. The selected input parameters for ENVI-met base simulation are shown in Table 1.

**Table 1:** Selected input parameters for ENVI-met base simulation.

**Category**	**Basic setting**
Wind speed measured in 10 m height (m/s)	7.9 m/s
Wind direction (0=N;90=E;180=S;270=W)	200
Relative humidity in 2 m	50%
Specific humidity at model top (2,500 mg/kg)	9 g/kg
Initial temperature of atmosphere (K)	306.15 K
Roughness length at measurement site	0.01
**Soil layer**	
Soil wetness upper layer (0–20 cm)	60%
Soil wetness middle layer (20–50 cm)	65%
Soil wetness deep layer (50–200 cm)	70%
Soil wetness bedrock layer (below 200 cm)	75%
Initial temperature upper layer (0–20 cm)	308
Initial temperature middle layer (20–50 cm)	310
Initial temperature deep layer (50–200 cm)	312
Initial temperature bedrock layer (below 200 cm)	314

**Figure 3:**
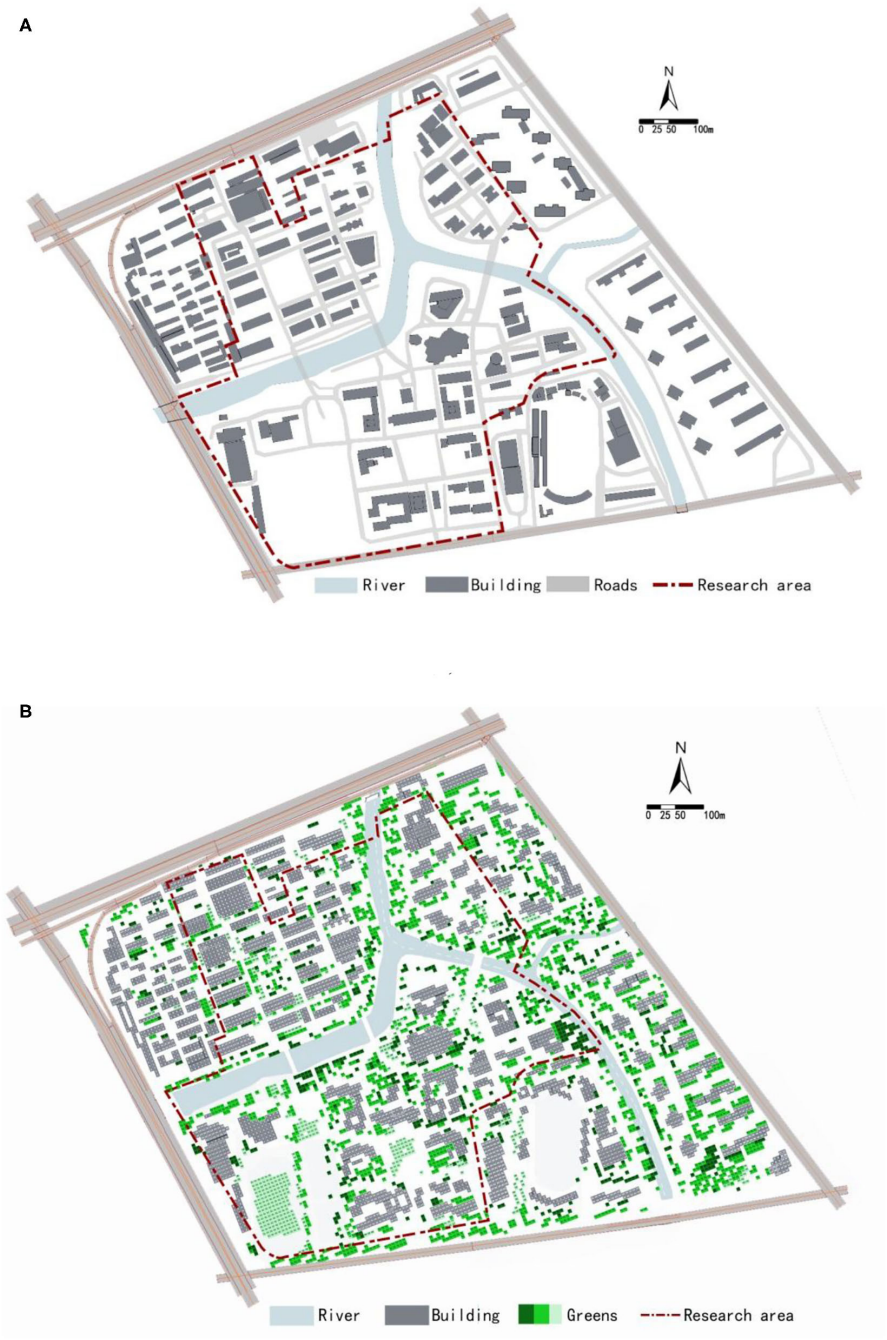
Simulation models: **(A)** NoiseSystem model, **(B)** ENVI-met model.

A simulation model is established based on the current situation of the Zhaohui campus of Zhejiang University of Technology (Figure 3). The physical environment in the pedestrian layer is assessed in models. The height of the pedestrian layer is 1.5 m. The NoiseSystem model and ENVI-met model set the height of the buildings, trees, and rivers in the two models as actual heights. Meanwhile, receiving stations at the same location are set for the evaluation of the simulation results (Figure 1B). A total of 133 receiving stations are laid out, with 119 evenly laid out on the campus and 14 off the campus, to have a more accurate understanding of the health-related physical environment of the campus. The date being simulated in this study is August 3, 2020, and the simulation starts at 10 am and lasts for 12 h. The horizontal section (with a height of 1.5) of the health-related physical environment at the pedestrian level is selected as the basis for simulation evaluation. Given that Hangzhou is dominated by the subtropical monsoon climate, the temperature at 2:00 p.m. is the daily high temperature in summer in Hangzhou. The daily high temperature has a significant influence on human comfort. Therefore, the data from 2:00 p.m. are used for each simulation.

### Evaluation Method

The health-related physical environment evaluation standards of different dimensions are selected, and evaluation indices are determined by referring to national standards and previous research results. The study also considers the probability values and types of the variables and quantifies the simulation and survey results of the health-related physical environment in the four dimensions of wind, thermal, acoustic and landscape to indicate the comfort level that the environment of different qualities brings to people. An 11-class linear scale is used for conversion to realize the normalization and dimensionless treatment of the indices (32). The scores are on a scale of 0 to 10.

### Acoustic Environment Evaluation

According to the category 0 indices in the Acoustic Environmental Quality Standards (GB3096-2008) (Supplementary Table 1), the simulation results of the acoustic environment at receiving stations in the study area are evaluated. The selected index is defined as the pass rate, that is, the area ratio of areas where the equivalent sound level meets the category 0 standard to the total area.

The UK's Planning Policy Guidance Note 24 divides Noise Exposure Categories (NEC) into four levels (34): A, B, C, and D (Supplementary Table 2). Level A indicates nearly no complaint about the impact of environmental noise, levels B and C indicate a certain degree of acceptance of the noise and Level D means that the noise is unacceptable. This guideline will be used to quantify the evaluation standard for the acoustic environment.

According to the four levels of Noise Exposure Categories (NEC), the disturbance of sound pressure levels is divided into four levels in the form of acceptance, and the level of acceptance is scored. In general, the levels of noise exposure in the university campus are mainly distributed in levels B and C. The levels of noise exposure correspond to the level of acceptance. Interpolation is used to determine the scores of acceptance levels 2 and 3 and distinguish the scores of the degree of annoyance caused by different sound pressure levels. We design wide ranges of scores of acceptance levels 2 and 3 to reveal the difference in the degree of annoyance caused by the slight difference among noise values. The quantitative evaluation of the acoustic environment is shown in Table 2A.

**Table 2:** Quantitative evaluation of different environment: **(A)** Acoustic Environment, **(B)** Wind Environment, **(C)** Thermal Environment.

**(A)**			
**Noise Exposure Categories (NEC)**	**Noise value (db)**	**Level of Acceptance (the degree of annoyance caused by different sound pressure levels)**	**Score (points)**
A	<55	Level 1, no annoyance	10
B	55–63	Level 2, little annoyance	5–10
C	63–72	Level 3, certain annoyance	0–5
D	>72	Level 4, severe annoyance	0
**(B)**			
**Beaufort Wind Scale (name of wind force)**	**Wind speed (m/s)**	**Level of Influence (the degree of influence of wind power level on human body)**	**Score (points)**
Level 0 (Calm)	0–0.2	Level 1, no feeling	0–4
Level 1 (Light air)	0.3–1.5	Level 2, not easy to detect	4–8
Level 2 (Light breeze)	1.6–3.3	Level 3, feeling bashing	8–10
Level 3 (Gentle breeze)	3.4–5.4	Level 4, a small amount of hair is blown away	4–8
Level 4 (Moderate breeze)	5.5–7.9	Level 5, hair is blown away and dust swirls in the air	0–4
Level 5 and above (Fresh breeze and above)	>8.0	Level 6, strong wind and difficult to walk	0
**(C)**			
**Predicted Mean Vote Scale**	**PMV value**	**Level of Thermal comfort (thermal comfort of human body with different PMV values)**	**Score (points)**
0	0~0.5	Level 1, comfortable	10
1	0.5~1.5	Level 2, warm	8–10
2	1.5~2.5	Level 3, hot	4–8
3	2.5~3.5	Level 4, very hot	0–4
4	>3.5	Level 1, extremely hot	0

### Wind Environment Evaluation

Simiu et al. (35) proposed a more specific relationship between pedestrian comfort and wind speed (Supplementary Table 3). Ng et al. (36) further subdivided the wind speed. Drawing on their 5-class wind speed classification standards, this study selects the area ratio of the windless zone as the basis for the wind environment evaluation. A wind speed of <0.6 m/s is used as the standard of the windless zone to evaluate the results of wind environment simulation in the study area.

The wind force scale devised by the British Francis Beaufort in 1805 is also known as the International Beaufort Wind Force Scale. According to this standard, China promulgated the national standard “Wind scale” (GB/T 28591-2012) (Supplementary Table 4). It further subdivides the wind force and wind speed ranges, with class 0 as calm, 1 as light air, 2 as a light breeze, 3 as a gentle breeze, 4 as a moderate breeze, and 5 as a fresh breeze. Starting from class 5, when the wind speed is >10 m/s, people will feel uncomfortable. Different wind forces and speeds have different effects on the human body. This scale will be used as an evaluation standard to quantify and grade the wind environment.

According to the Beaufort Wind Force Scale, the impact of wind speed on the human body is divided into six levels in terms of how much the wind affects the human body. The wind speed that is between 1.6 and 3.3 m/s provides the most comfortable wind environment for people, so the assigned score is the highest. We design the wide ranges of scores of acceptance levels 0, 1 3 and 4 to reveal the difference in the degree of the influence of wind power level on the human body caused by the slight difference in wind speed. The impact is graded as follows: calm, level 1, 0–4 points; light air, level 2, 4–8 points; the light breeze, level 3, 8–10 points; gentle breeze, level 4, 4–8 points; the moderate breeze, level 5, 0–4 points; and strong breeze and above, level 6, 0 points. Interpolation is used to determine the score for impact levels 1 to 5 (Table 2B).

### Thermal Environment Evaluation

China's Green Building Evaluation Standards (GB 50378-2006) propose that the daily average outdoor heat island intensity of residential areas should not be higher than 1.5°C. The selected index is the area ratio of the high-temperature zone. According to the standard of measured average heat island intensity in summer ≤ 1.5°C, this study regards the area ratio of the area where the Mean Radiant Temp. (MRT) exceeds the average of receiving stations by 1.5°C as the area ratio of the high-temperature zone and evaluates the simulation results of the thermal environment at receiving stations in the study area.

Compared with the other thermal comfort indices, the PMV index proposed by Fanger considers factors such as human body parameters, body metabolic rate, and clothing insulation coefficient in detail (18). The thermal sensation of the human body can generally be rated using the ASHRAE 7-point scale and its extended 9-point scale (37). The two scales are symmetrical. Point 0 on the scale indicates that people feel neither too hot nor too cold, which is defined as thermal neutrality. The three points in the middle of the scale, namely, −1, 0, and 1, are considered satisfactory. The PMV evaluation model in this work is built according to the ASHRAE extended 9-point scale (Supplementary Table 5). For the PMV values between −0.5 and 0.5, the outdoor thermal environment is the most favorable. Therefore, it will be used as the evaluation standard to quantify the thermal environment.

Given that this study simulates a day in summer, all PMV values are positive. Considering the points greater than 0 on the ASHRAE 9-point scale, the PMV value is divided into five levels in the form of thermal comfort, which is graded (Table 2C). This method of assignment for PMV is similar to the method of assignment for the acoustic environment.

### Landscape Environment Evaluation

Different types of landscape spatial forms, including riverside space, buildings, greening and squares, are present in the study area. This study uses questionnaire surveys to obtain data for evaluating the visual satisfaction of different forms of landscape environment and determining the level of landscape perception in each area. Sampling units in this study are individuals over 18 years old. All teachers and students on the Zhaohui campus of Zhejiang University of Technology are included in the sample population. The questionnaire consists of two parts: the first part is the socio-demographic characteristics of the respondents, and the second part is their spatial perception and preference scores for landscape elements. The questionnaire for landscape perception evaluation is shown in Table 3. The evaluative dimension includes spatial characteristics, environment greening, and psychological perception, and an 11-class linear scale is used for conversion (38, 39).

**Table 3:** The questionnaire for Landscape perception evaluation.

**Evaluative dimension**	**Evaluation factor**	**Adjective**	**Grading range(0–10 scores)**											**Adjective**
			**Very poor**→**Very Good**											
			**0**	**1**	**2**	**3**	**4**	**5**	**6**	**7**	**8**	**9**	**10**	
Spatial characteristics	Walkability	Poor suitability												Good suitability
	Color matching	Unreasonable												Reasonable
Environment greening	Plant richness	Simple												abundant
	Environmental coordination	Incongruous												Congruous
Psychological perception	Novelty	Dull and boring												Novel and interesting
	Security	Dangerous												Safe

### Overall Evaluation of the Area

The final step of the analysis is to estimate the score based on the weighted sum of the four categories of environment. The equal weight method for calculating the overall score is widely used in comprehensive assessment (40, 41). It assumes that different influencing factors have the same impact on the evaluation object. Therefore, the equal weight method is used in this study, and a weight of 25% is given to each type of environment since the importance of the impact of each environment on the human body is similar.


$$\begin{array}{l} OSA=\sum \left(P_{i}\times W_{i}\right) \end{array}$$

where *OSA* is the overall score of the whole area, *P*_*i*_ is the score of each type; and *W*_*i*_ is the weight of each type (25%).

## Results

### Performance Evaluation

**Figure 4:**
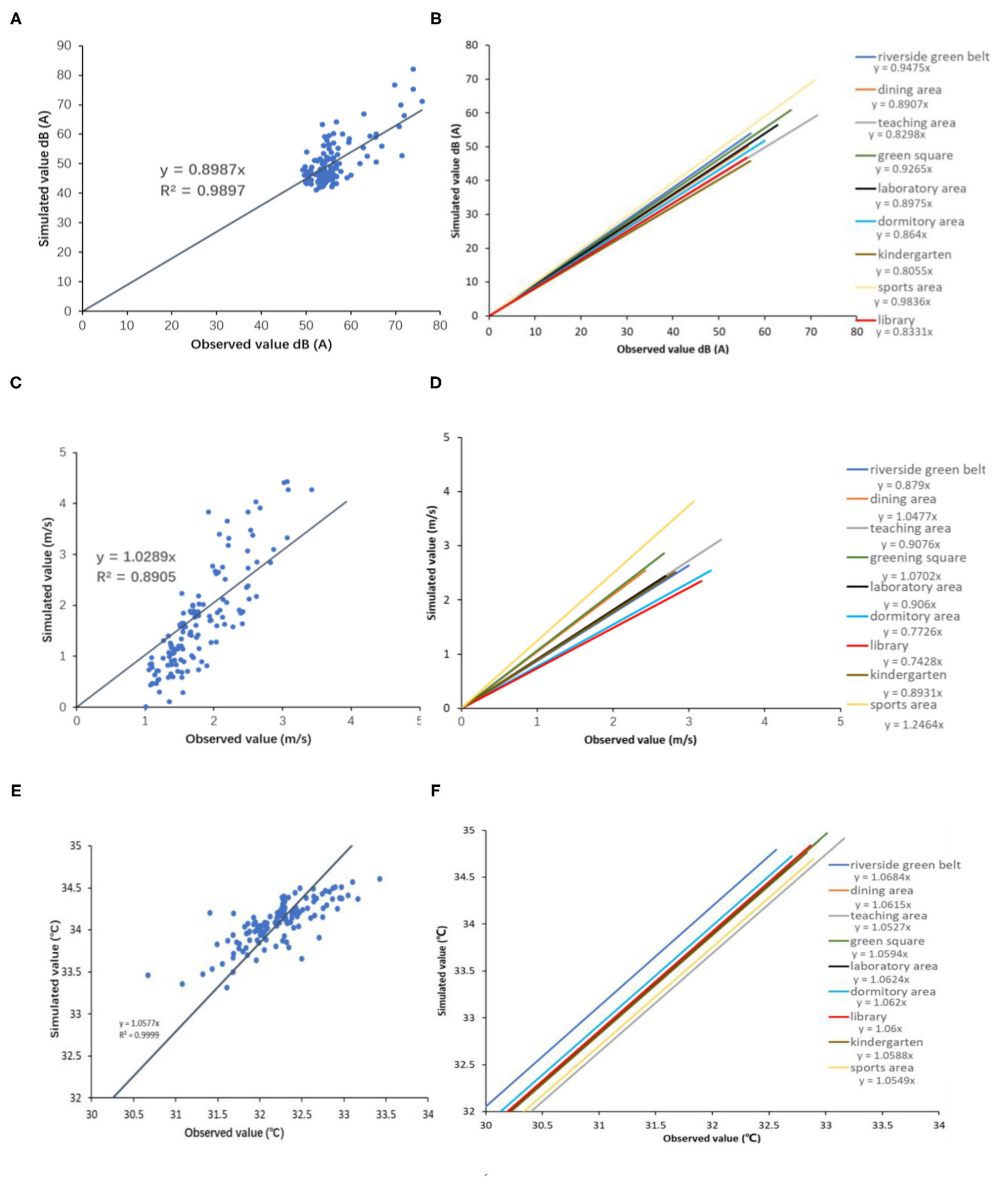
Curves of simulated value and observed value: **(A)** Performance tests of acoustic environment in whole area, **(B)** Performance tests of acoustic environment in separated areas, **(C)** Performance tests of wind environment in whole area, **(D)** Performance tests of wind environment in separated areas, **(E)** Performance tests of thermal environment in whole areas, **(F)** Performance tests of thermal environment in separated areas.

We compare the simulated data against the observed *in situ* data to test the model performance. The differences between simulated and observed values of equivalent A sound level, wind speed, and average radiation temperature in the whole campus and control points of different functional zones are assessed. Figure 4 reveals that the values of *R*^2^ of linear fitting equations for the values of equivalent A sound level and average radiation temperature are larger than 0.95, and the values of *R*^2^ of the linear fitting equation for wind speed are near 0.90. The model performance implies that the model can be applied to simulate the health-related physical environment.

### Health-Related Physical Environment Simulation

Based on the simulation results of the three types of the health-related physical environment (wind, thermal and acoustic), wind speed, temperature, humidity, and equivalent A sound level were measured at the 133 receiving stations in the study area. Moreover, spatial raster analysis was performed on the wind speed, temperature, and equivalent A sound level values of the receiving stations by using ArcGIS software. A 100 × 100 grid was created for the study area, and the resulting space obtained was linked to different functional areas. The area ratio of the windless zone, the area ratio of the high-temperature zone and the equivalent A sound level pass rate of each area were calculated.

### Acoustic Environment

**Figure 5:**
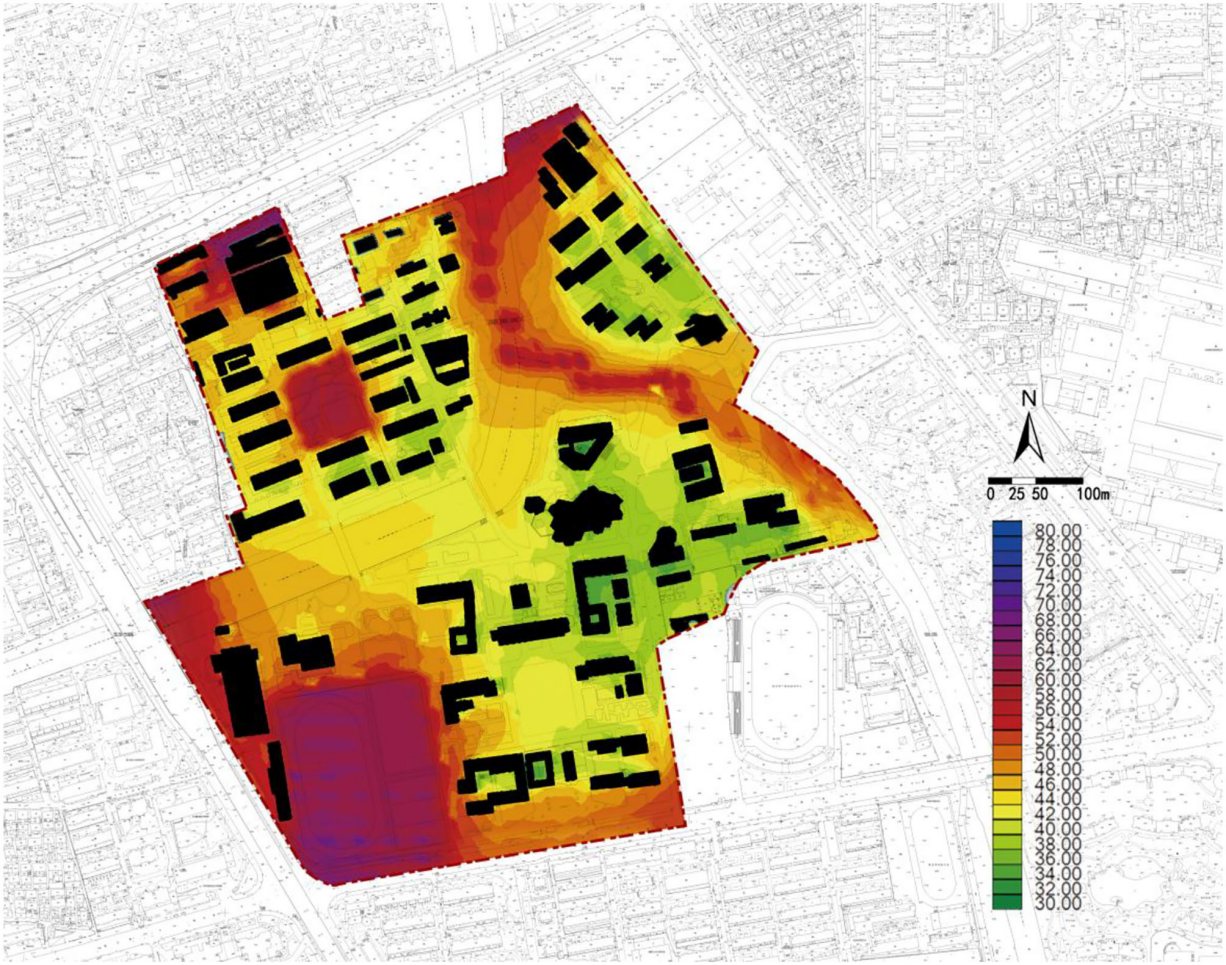
Zhaohui Campus of Zhejiang University of Technology: Numerical simulation diagram of equivalent A sound level at 14:00.

The acoustic environment simulation results (Figure 5) show that the equivalent A sound level in the entire area falls within 41–67 db, indicating a generally good acoustic environment on the campus. However, a distinct difference is observed between areas with a good and poor acoustic environment. The equivalent A sound level value is higher near the elevated roads to the north and the west, in the sports courts, and along the river. The reason is that most of these places are in the exposed area. By contrast, the acoustic environment of other areas protected by buildings and plants is relatively better, especially in areas in the east and southeast, which are sheltered by high-rise buildings outside the boundary and have rich greenery in the area. Thus, the equivalent sound level is low at under 50 db.

In terms of each functional area, Table 4 shows that the overall equivalent A sound level of each area is relatively high, with the pass rate of most areas over 50%. Figure 5 shows that areas with unqualified equivalent A sound levels are the sports area, green square 1, and laboratory areas. The pass rates of the three areas are 11.44, 14.02, and 28.92%, respectively.

**Table 4:** Pass rate of Average Equivalent Sound Level (AESL) in each zone.

**Zone**	**Pass rate of AESL (%)**	**Zone**	**Pass rate of AESL (%)**	**Zone**	**Pass rate of AESL (%)**	**Zone**	**Pass rate of AESL (%)**
Laboratory area	28.92	Dining area 2	93.22	Dormitory area 4	97.78	Green square 3	100.00
Dining area 1	68.57	Dormitory area 3	79.70	Kindergarten	100.00	Teaching area 2	69.71
Dormitory area 1	89.87	Riverside green belt 1	64.63	Sports area	11.44	Teaching area 3	100.00
Dormitory area 2	78.52	Riverside green belt 2	71.51	Green square 2	83.82	Library	100.00
Green square 1	14.02	Dining area 3	52.43	Teaching area 1	73.98	Green square 4	100.00

### Wind Environment

**Figure 6:**
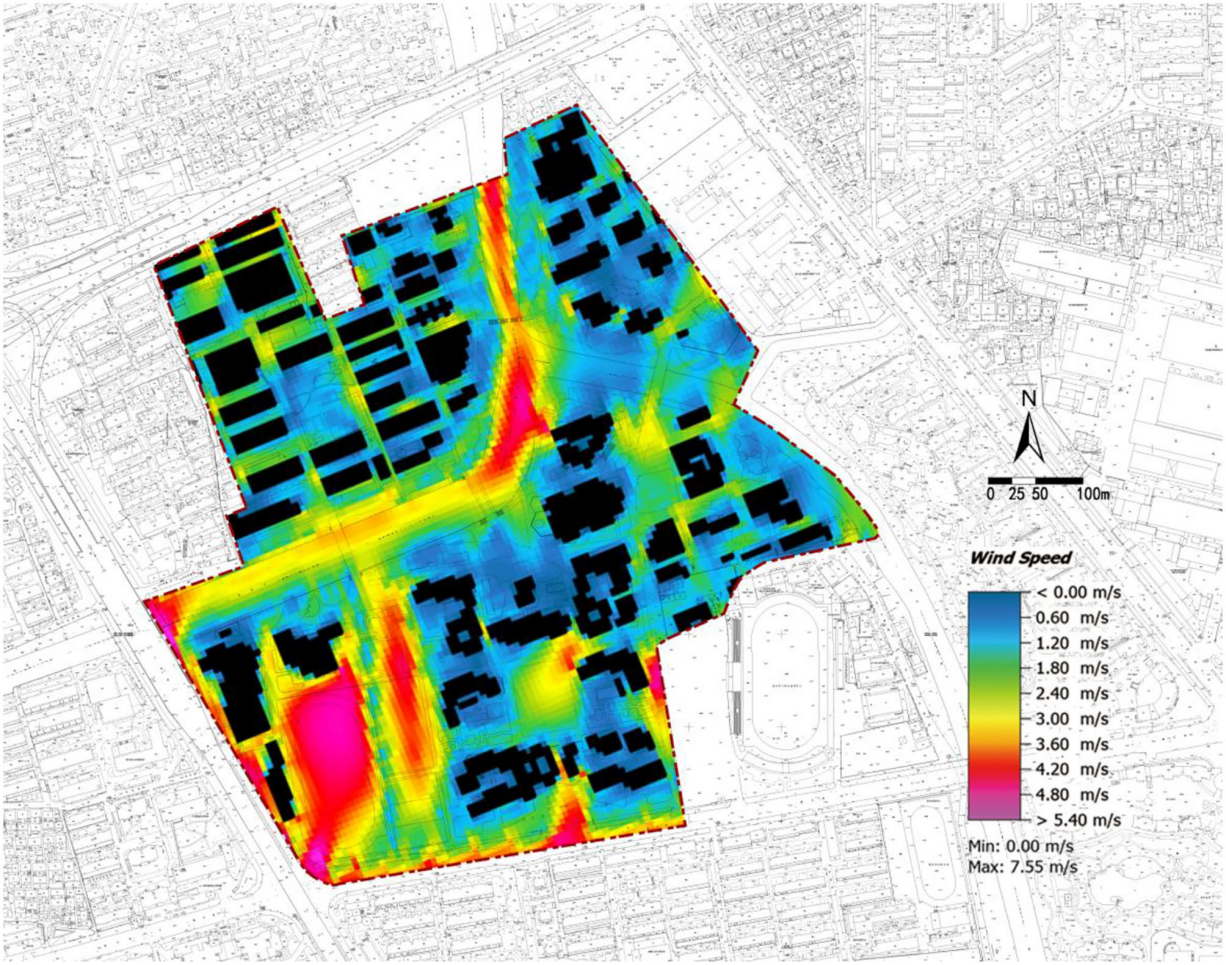
Zhaohui Campus of Zhejiang University of Technology: Numerical simulation diagram of wind speed at 14:00.

The wind environment simulation results (Figure 6) show that the wind speed in the area is generally lower than 5 m/s, and the overall wind environment is comfortable. Dense buildings will create a light breeze/air and windless zone of the considerable area with a wind speed lower than 2 m/s, especially when the areas are surrounded and enclosed by buildings. However, the wind speed in a small number of areas, mainly in the sports area, exceeds 5 m/s.

According to the evaluation standard of the windless zone with wind speed lower than 0.6 m/s, the area ratio of the windless zone for each area is calculated based on the simulation results (Table 5). The results show that the area ratio of the windless zone falls within 1.4–28.31%. Figure 6 shows that a windless zone, including teaching areas 1 and 2, the library, and dormitories 3 and 4, will be formed around buildings. The windless area ratios of teaching area 1 and dormitory area 4 are larger than 20%. Teaching area 1 has the largest windless area ratio of 28.31%. The thermal comfort in these areas is poor.

**Table 5:** Area ratio of Windless Zone (WZ) in each zone.

**Zone**	**Area ratio of WZ (%)**	**Zone**	**Area ratio of WZ (%)**	**Zone**	**Area ratio of WZ (%)**	**Zone**	**Area ratio of WZ (%)**
Laboratory area	2.06	Dining area 2	6.64	Dormitory area 4	22.12	Green square 3	4.10
Dining area 1	1.40	Dormitory area 3	18.37	Kindergarten	15.79	Teaching area 2	14.26
Dormitory area 1	7.36	Riverside green belt 1	6.28	Sports area	1.39	Teaching area 3	13.79
Dormitory area 2	8.23	Riverside green belt 2	10.41	Green square 2	12.72	Library	17.87
Green square 1	12.12	Dining area 3	6.16	Teaching area 1	28.31	Green square 4	8.69

### Thermal Environment

**Figure 7:**
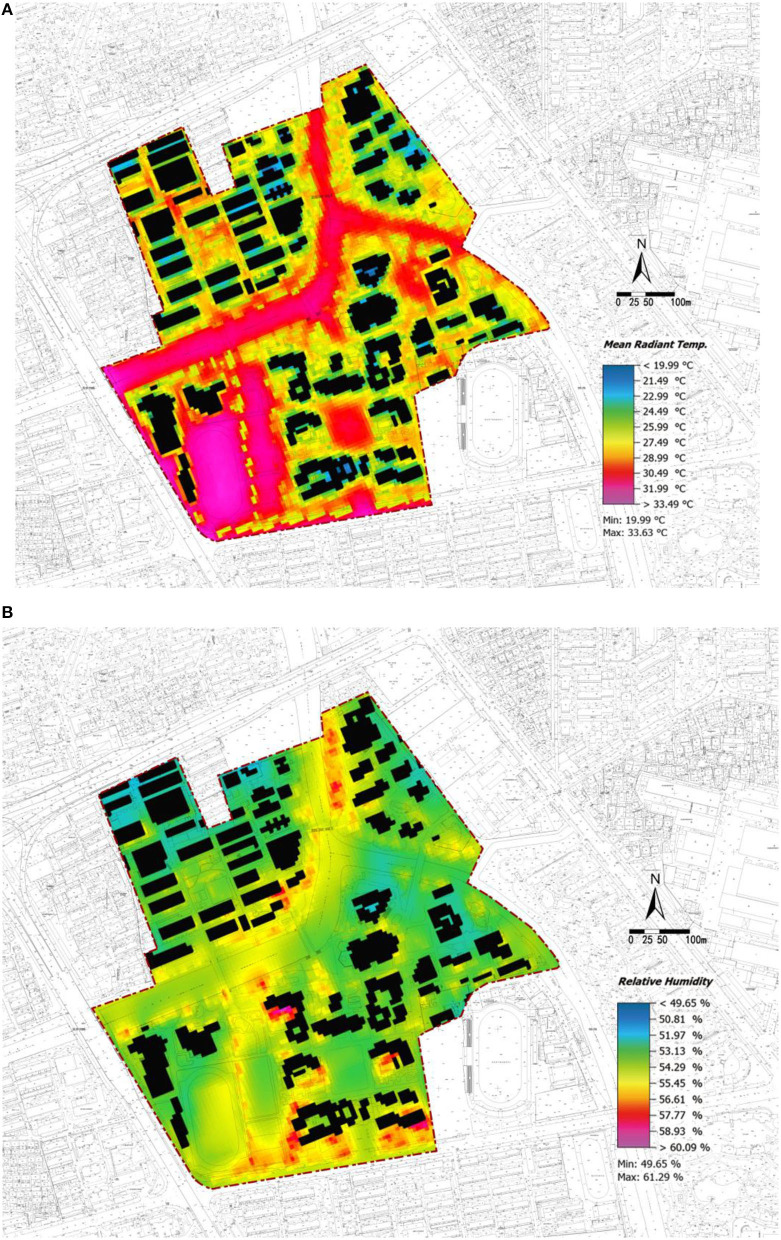
Zhaohui Campus of Zhejiang University of Technology: Numerical simulation diagram at 14:00: **(A)** Mean Radiant Temp. (MRT), **(B)** Relative Humidity.

Thermal comfort is considered to be directly related to health and work efficiency and is closely related to climatic conditions such as temperature and humidity, which are important factors affecting the thermal environment. The average radiant temperature and relative humidity obtained from the simulation are selected for analysis. The summer Mean Radiant Temp (MRT) range to achieve thermal comfort is 17–26°C. However, the simulation results (Figure 7A) show that the Mean Radiant Temp. (MRT) is >26°C for most places in the study area. Therefore, the temperature in the whole area is generally high, and high-temperature zones may be formed (close to rivers and the sports area). Given that the lowest relative humidity is 49.65% and the highest is 61.29% (Figure 7B), the overall humidity is relatively high. Accordingly, the human body cannot lose heat and people will feel hot and suffocating.

In terms of the thermal environment of each area, the results show (Table 6) that the sports area has the greatest high-temperature zone ratio of 87.44%, followed by green square 2 of 54.69%. The high-temperature zone ratio of the sports area and green square 2 is larger than 50%. High-temperature zones are easily formed in the two areas due to their large proportion of hard-surfaced pavements that lack water permeability and air permeability.

**Table 6:** Area ratio of High-temperature Zone (HTZ) in each zone.

**Zone**	**Area ratio of HTZ (%)**	**Zone**	**Area ratio of HTZ (%)**	**Zone**	**Area ratio of HTZ (%)**	**Zone**	**Area ratio of HTZ (%)**
Laboratory area	4.90	Dining area 2	0.17	Dormitory area 4	0.32	Green square 3	17.07
Dining area 1	1.43	Dormitory area 3	0.23	Kindergarten	0.57	Teaching area 2	3.91
Dormitory area 1	0.21	Riverside green belt 1	0.68	Sports area	87.44	Teaching area 3	3.16
Dormitory area 2	0.23	Riverside green belt 2	0.58	Green square 2	54.69	Library	1.60
Green square 1	2.80	Dining area 3	0.19	Teaching area 1	10.20	Green square 4	0.76

### Subjective Evaluation of Landscape Environment

This study uses a random sampling method and randomly selects five people from each of the 20 areas to collect data. The questionnaire survey was performed face-to-face on campus from August 1 to 3, 2020. The survey first investigated the socio-demographic characteristics of the interviewees, and then the interviewees were asked to compare the subjective visual perception of the existing landscape environment in each area and assign a score from 0 to 10 to determine the level of landscape perception. The participant took about 10 min to complete the questionnaire. Most of the respondents are male, aged 21 to 35 years, and 39% of them are faculty and staff. Data from 108 interviewees were collected, and the effective rate of the survey was 92.6%. It will be used for the study of the quantitative evaluation of landscape environment perception.

The results show that a great number of green belts and areas are adjacent to the river in the study area, such as riverside green belts and green squares. These areas usually give people a better visual perception of the landscape, and the corresponding perception level of the landscape is also high. However, the landscape perception level of laboratory areas and the sports area is lower. The overall landscape perception level of dormitories and dining areas is generally lower than that of the teaching area.

### Integrated Evaluation of Health-Related Physical Environment

**Figure 8:**
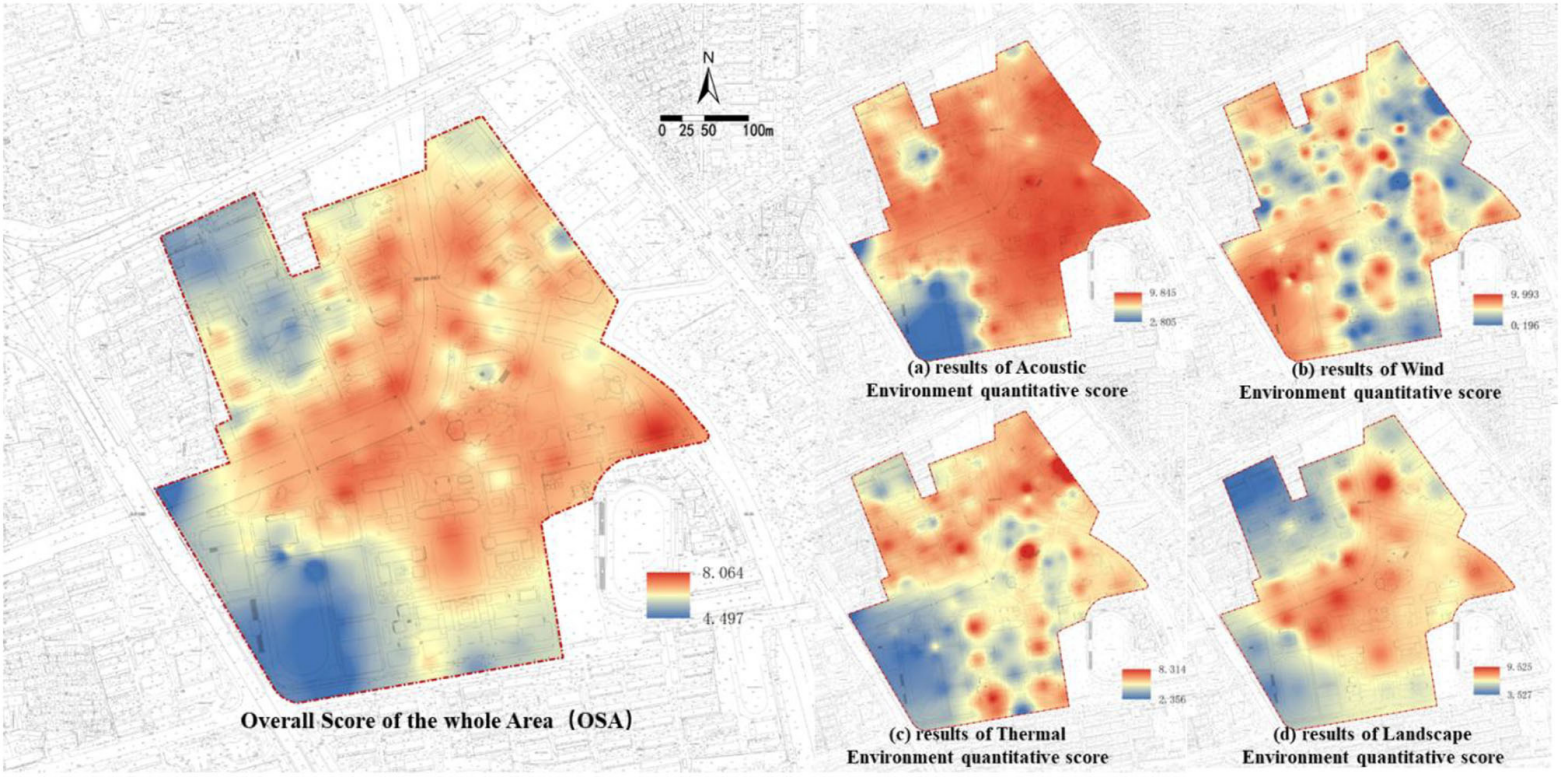
Spatial visualization of quantified scoring results in health-related physical environment.

**Figure 9:**
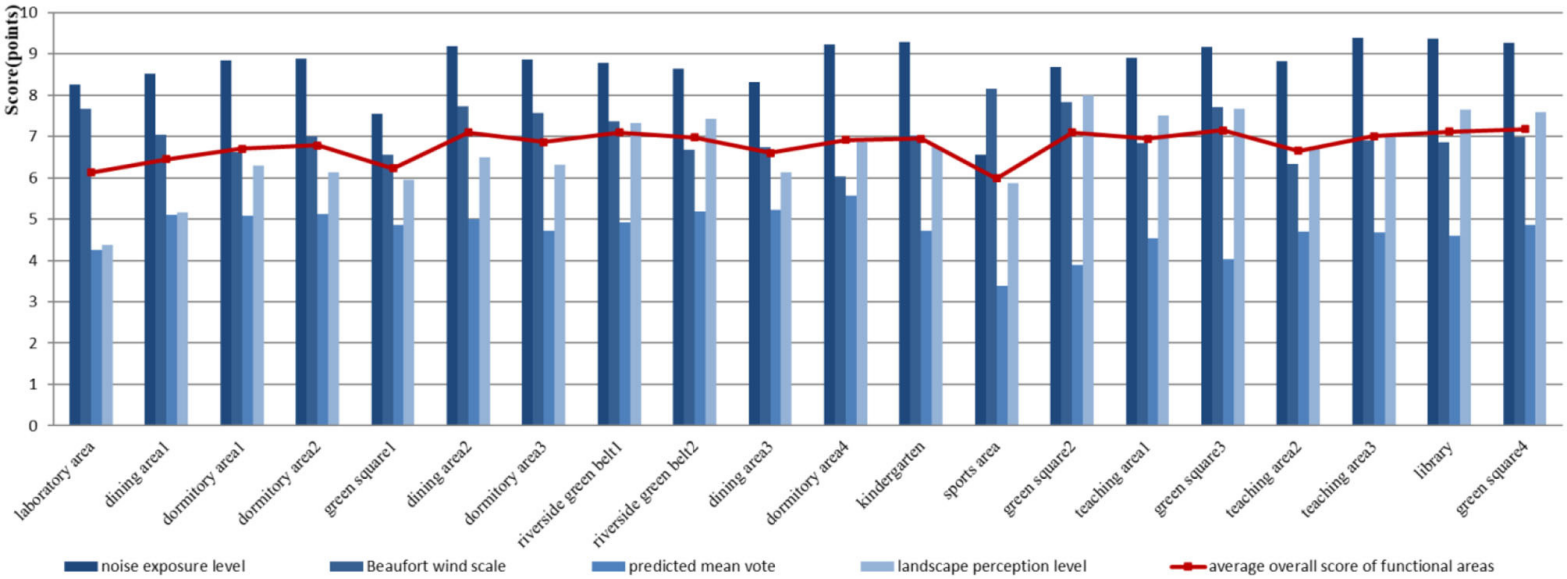
Quantitative scoring results of the health-related physical environment in each zone.

The four dimensions of the health-related physical environment are quantified and graded according to the given evaluation standards, and the scores are then visualized. ArcGIS software is used to perform spatial raster analysis on the evaluation values of the receiving stations to obtain the spatial perception distribution of the health-related physical environment in each dimension. The integrated score of the whole area is obtained by the weighted sum of the four types of health-related physical environments by using a raster. The result is shown in Figure 8. Similarly, a 100 × 100 grid for the study area is created, and the resulting space is linked after rasterization to different functional areas. The average scores of the noise exposure level, Beaufort wind force scale, thermal comfort PMV and landscape perception level are calculated, as shown in Figure 9.

The average scores of each area (Figure 9) show that the thermal environment is the worst among the four types of the health-related physical environment. The PMV score of thermal comfort is relatively low. The acoustic environment is the best, followed by the wind environment. The score of the landscape perception level fluctuates greatly, and distinct differences are observed among the areas. Hence, thermal comfort is the worst in the four types of environments in summer. The bother caused by noise is slight. Wind comfort and visual acceptance of landscape are different in different regions. Combining the visualization results in Figure 8 and the acoustic environment results (Figure 8A) shows that the acoustic environment of the entire area is satisfactory with the average score of the noise exposure level above 8 except for the sports area and green square 1. The score of the sports area is lower than 7. Figure 8B shows that the distribution of areas with a low comfort score in terms of the wind environment conforms with that of windless zones mentioned in the previous section. Figure 9 shows that the average score of the wind environment falls within 6.027–8.154, indicating an overall satisfactory environment. Figure 8C shows that the areas with high thermal sensation scores are the two large dormitories in the north and teaching area 3 in the west. Figure 9 shows that the sports area has the lowest average score of only 3.375 in terms of the thermal environment. The PMV score of sports area and green squares 2 is lower than 4. The average scores of the other areas are around 5, which means that the thermal environment is unsatisfactory. The sports area and green squares 2 and 3 have higher scores in terms of the wind environment, while the opposite is observed for the thermal environment. The landscape perception scores and spatial visualization (Figures 8D, 9) show that areas with low landscape environment scores are the sports area (5.878), the laboratory area in the north (4.365), and dining area 1 (5.163). The levels of landscape perception in these areas are low.

The broken line graph in Figure 9 shows that the sports area has the lowest average overall score among all functional areas, followed by laboratory areas. The visualization results of the scores of all areas on campus in Figure 8 show that the library and the areas located within a certain range along both sides of the river have higher scores. Meanwhile, the areas close to roads in the west and north (sports area and south of teaching area 2) and laboratory areas and surrounding areas under their influence (green square 1) have lower scores.

## Discussion

The acoustic environment relates to human health. Noise has an impact on hearing health (22). Most of the sports areas and laboratory areas are in the exposed area and are affected by the types of activities happening inside them and noise originating from outside highways. Residents who stay in these areas for a long time suffer the risks of hearing loss and mental stress. Okokon et al. (1) found that road-traffic noise can induce stress, which may contribute to mental health disorders. Excessive noise in the physical environment in urbanizing areas is related to mental illness, heart disease, stress, sleep quality, and cognitive impairment (21). Alleviating the noise influence in the physical environment should improve the acoustic environment.

Wind environment relates to human health. A windless zone in this study, such as teaching areas, library, and dormitories, will be formed around buildings. The windless zones formed in these areas are not conducive to the dispersion of air pollutants and increase respiratory health risks (42). Meanwhile, the relative thermal discomfort of these windless zones due to the enclosed space created by buildings and small ventilation corridors resulting from dense buildings may lead to the heat island effects in part of the campus (5). Therefore, these areas should be the key areas in the improvement of the wind environment quality. Possible improvement measures include adjusting the architectural layout at air passages, adding ventilation corridors and connecting to surrounding open spaces (e.g., squares and rivers) and peripheral air flow channels (e.g., roads) for introducing airflow into the building complex and promoting the internal air flow. It can reduce the accumulation of pollutants and reduce health risks, such as respiratory diseases caused by insufficient ventilation.

The thermal environment relates to human health. A significant correlation exists between temperature and thermal comfort (43). Higher temperature corresponds to a lower PMV score. Shadow areas that can be formed by buildings or plants are inadequate because of the exposed areas, such as sport areas, lack projection of buildings and plants. The water and air impermeability of the hard-surfaced pavements results in a low score in terms of the thermal environment. It is prone to skin diseases or heatstroke and other diseases in summer, and its thermal comfort is poor. Liu et al. (44) found that heat index and air pollution index are significantly associated with mortality. The combination adjustment of temperature, humidity, and wind speed can improve thermal comfort (45). Thus, the improvement of the quality of the wind and thermal environment and cooling measures should be proposed to increase the human comfort level and reduce the risks of heat stroke.

The landscape environment relates to human health. The landscape has a significant impact on human health. The overall landscape perception level of dormitories and dining areas is generally lower than that of the teaching area because hard-surfaced roads and dilapidated buildings can cause anxiety and irritability. Thus, the improved visual quality caused by landscape can improve physiological and mental comfort (46, 47).

Integrated evaluation of the health-related physical environment can determine the most unsatisfactory environment in a certain area. From the perspective of improving public health, this study helps to design an optimization strategy to improve the health-related physical environment (48, 49). The strategy needs to focus on the sports area in the south and the northernmost laboratory area considering the thermal environment scores. This method can also be used to determine the type of health-related physical environment with the lowest score for revealing the improvement strategy. For example, when attention is paid to the improvement of thermal environment quality for laboratory areas, the visual quality of the landscape should also be considered. For teaching area 2, the improvement of wind and thermal comfort and the visual quality of the landscape should be conducted simultaneously. Laboratory areas have an overall low score. Thus, improvement should be performed comprehensively on the four types of environment. Improvement for the sports area should include installing noise barriers to improve the quality of the acoustic environment and partial greening or adding rest corridors to enhance the quality of the thermal environment and landscape environment perception.

## Conclusions

### Major Conclusions and Optimization Suggestions

This study proposes a method for investigating health-related physical environment in urbanizing areas. The method integrates subjective and objective evaluation to comprehensively assess the multi-dimensional physical environment. This method combines health-related physical environment simulation technology with GIS spatial analysis to identify priority intervention areas, which can be used to support spatial optimization decision-making in urban planning and design. The method also serves as a pre-evaluation work for the early stages of urban design and provides action plans for optimizing the urban form and improving the poor comfort and high incidence of health diseases caused by environmental degradation. This method is applied to a case study of an urbanizing area (Zhaohui Campus of Zhejiang University of Technology). The results highlight the five major optimization suggestions for the area. (1) The implementation of ventilation corridors needs to be strengthened, especially in the teaching area, the library, and the dormitory area on the east side to reduce the area of windless zones for improving the wind environment. This strategy should improve wind comfort, promote air flow in enclosed spaces and reduce the high risk of respiratory diseases. (2) Cooling measures are proposed to mitigate the heat island effect in high-temperature areas such as the sports area and the two green squares in the south for improving the thermal environment. This method should improve thermal comfort and reduce health risks associated with skin diseases, heatstroke, and cardiovascular and cerebrovascular diseases. (3) Noise barriers should be installed, especially in the sports area and laboratory areas, to improve the acoustic environment. This proposal should reduce the annoyance of noise and prevent mental health, hearing health, sleep disorder, and other diseases caused by noise. (4) Attention should be paid to improving the landscape visual quality of the sports area, laboratory areas, and dormitories in the north to enhance the landscape environment. Attention should be paid to the design of a healthy landscape and improving the visual perception of landscape to promote residents' psychological, emotional and physiological health. (5) The sports area, laboratory areas, and green square 1 should be regarded as key areas for the multi-dimensional comprehensive improvement of the environment. All the four dimensions of the health-related physical environment need to be strengthened.

### Innovation and Limitations

This study is innovative mainly in three aspects. (1) It performs the multi-dimensional evaluation of the four types of the health-related physical environment of acoustic, wind, thermal, and landscape under multi-factor action mechanisms of wind speed, temperature, noise, and landscape perception to examine and simulate the health-related physical environment. (2) It introduces subjective indices, which can more accurately reflect human feelings, and conduct a comprehensive survey of the physical environment's influence on human comfort level and health risk through the combination of subjective and objective evaluations. (3) It performs pre-evaluation on the health-related physical environment, proposes spatial optimization strategies, and provides action plans for the next steps of urban design.

However, the limitations must be acknowledged. First, the indices used in the evaluation of simulation results are the equivalent sound pressure level, wind speed, and average radiation temperature. The maximum and minimum sound pressure levels are ignored in the simulation analysis. Second, errors exist in the model construction and the quantity and quality of data measured at receiving stations, thereby affecting planning and decision making. For future research, an increase in the number of input variables or evaluation categories for larger data sets will be considered. In addition, establishing cooperation with institutions from complementary research fields may be considered in the future to overcome data limitations. Finally, we cannot run the models again to assess and ascertain the potential improvements of the suggestions, which are difficult to be quantified. Meanwhile, this study pays more attention to the present situation evaluation, and the optimization simulation is considered in future research.

## Data Availability Statement

The raw data supporting the conclusions of this article will be made available by the authors, without undue reservation.

## Ethics Statement

Ethical review and approval was not required for the study on human participants in accordance with the local legislation and institutional requirements. Written informed consent for participation was not required for this study in accordance with the national legislation and the institutional requirements.

## Author Contributions

YW, HY, and YG conceived of the presented idea and supervised the findings. SiW, XQ, ShW, SY, and WL collected data and performed the computations. YW, SiW, XQ, ShW, SY, WL, HY, JZ, SX, and YG wrote the original manuscript. YW, SiW, HY, JZ, SX, and YG revised them critically. All authors have made a substantial and intellectual contribution to this article and approved it for publication.

## Funding

This research received financial support from the Key Project of National Social Science Foundation of China (21FGLA002), the National Natural Science Foundation of China (51578507 and 71874151), the Industry-University Cooperation Collaborative Education Project of Ministry of Education of China (201902112026), and the Zhejiang Provincial Natural Science Foundation of China (LZ22G030005).

## Conflict of Interest

The authors declare that the research was conducted in the absence of any commercial or financial relationships that could be construed as a potential conflict of interest.

## Publisher's Note

All claims expressed in this article are solely those of the authors and do not necessarily represent those of their affiliated organizations, or those of the publisher, the editors and the reviewers. Any product that may be evaluated in this article, or claim that may be made by its manufacturer, is not guaranteed or endorsed by the publisher.
