# Supplementary material for: Integrated Evaluation Method of the Health-Related Physical Environment in Urbanizing Areas: A Case Study From a University Campus in China
Source: Front Public Health. 2022 Feb 8;10:801023. doi: 10.3389/fpubh.2022.801023 (PMC8861282; doi:10.3389/fpubh.2022.801023)
Supplement: Supplementary file 1 [file Data_Sheet_1.docx]

Supplementary Material

**Supplementary Table 1.** Environmental noise limit dB(A)

| Acoustic environment functional zones category | | Time | |
| --- | --- | --- | --- |
|  |  | Daytime | Nighttime |
| Class 0 | | 50 | 40 |
| Class 1 | | 55 | 45 |
| Class 2 | | 60 | 50 |
| Class 3 | | 65 | 55 |
| Class 4 | Class 4a | 70 | 55 |
|  | Class 4b | 70 | 60 |

- Class 0 of acoustic environment functional zones category: Refers to areas in need of quiet, such as rehabilitation areas.
- Class 1 of acoustic environment functional zones category: Refers to areas where residential housing, medical care, cultural education, scientific research design, and administrative offices are the main functions that need to be kept quiet.
- Class 2 of acoustic environment functional zones category: Refers to areas where commercial finance and market trade are the main functions, or where residence, commerce, and industry are mixed, and residential quiet areas need to be maintained.
- Class 3 of acoustic environment functional zones category: Refers to areas with industrial production, warehousing and logistics as their main functions, and the need to prevent industrial noise from having a serious impact on the surrounding environment.
- Class 4 of acoustic environment functional zones category: Refers to the area within a certain distance on both sides of the main road, which needs to prevent the traffic noise from having a serious impact on the surrounding environment, including two types of 4a and 4b.Category 4a is the area on both sides of highways, first class highway highways,secondary highways, urban expressways, urban trunk roads, urban secondary roads, urban rail transit (ground section), and inland waterways; Category 4b is the area on both sides of the railway trunk line.

**Supplementary Table 2.** PPG 24 recommends various noise exposure level limits for homes with nearby noise sources

| Noise source | Period | Noise Exposure Categories (NEC) | | | |
| --- | --- | --- | --- | --- | --- |
|  |  | A | B | C | D |
| Road traffic noise | 07：00~23：00 | <55 | 55~63 | 63~72 | >72 |

Guidance, P. P. (1994). 24 (PPG24)–Planning and Noise. Department of the Environment.

**Supplementary Table 3.** The impact of pedestrians by wind

| Wind Speed m/s | Human feeling |
| --- | --- |
| 1-5 | Comfortable |
| 5-10 | Uncomfortable, movement affected |
| 10-15 | Very uncomfortable, movement is severely affected |
| 15-20 | Can not stand |
| >20 | Dangerous |

Scanlan, R. H. (1986). Wind effects on structures: an introduction to wind engineering. Wiley.

**Supplementary Table 4.** Wind scale rating

| Beaufort scale | Name of wind force | 10m wind speed above the ground on flat ground (m/s) | Terrestrial ground phenomenon |
| --- | --- | --- | --- |
| 0 | Calm | 0-0.2 | Quiet, smoke straight up |
| 1 | Light air | 0.3-1.5 | Smoke shows wind direction |
| 2 | Light breeze | 1.6-3.3 | Feel windy |
| 3 | Gentle breeze | 3.4-5.4 | Flag unfolding |
| 4 | Moderate breeze | 5.5-7.9 | Blow up the dust |
| 5 | Fresh breeze | 8.0-10.7 | Small tree swing |
| 6 | Strong breeze | 10.8-13.8 | Wires sound |
| 7 | Near gale | 13.9-17.1 | Difficulty walking |
| 8 | Gale | 17.2-20.7 | Destroy the branches |
| 9 | Strong gale | 20.8-24.4 | Small damage to the house |
| 10 | Storm | 24.5-28.4 | Uproot trees |
| 11 | Violent storm | 28.5-32.6 | Severe damage |
| 12 | Hurricance | 32.7-36.9 | Destroy greatly |

Reference: China National Standard《Wind scale》（GB/T 28591-2012）

**Supplementary Table 5.** Thermal comfort PMV evaluation model

| Thermal sensation vote | ASHRAE scaling | PMV value |
| --- | --- | --- |
| Extremely cold | -4 | <-3.5 |
| Very cold | -3 | -3.5~-2.5 |
| Cold | -2 | -2.5~-1.5 |
| Cool | -1 | -1.5~-0.5 |
| Comfortable | 0 | -0.5~0.5 |
| Warm | 1 | 0.5~1.5 |
| Hot | 2 | 1.5~2.5 |
| Very hot | 3 | 2.5~3.5 |
| Extremely hot | 4 | >3.5 |

Note: Fanger, a famous Danish professor, pointed out the bio-meteorological index of PMV human thermal comfort after analyzing the physical environment factors and people's physical comfort factors, which represents the average value of cold and hot comfort of most people in the same physical environment.
